# Bleeding and Thrombotic Challenges in Children with uncorrected Cyanotic Congenital Heart Diseases (CCHD)- Experience from a tertiary care pediatric hospital

**DOI:** 10.12669/pjms.41.11.12609

**Published:** 2025-11

**Authors:** Nazish Saqlain, Tehmina Kazmi, Munawar Ghous, Masood Sadiq

**Affiliations:** 1 Nazish Saqlain, FCPS (Hematology) Department of Hematology and Transfusion Medicine, University of Child Health Sciences, The Children’s Hospital, Lahore, Pakistan; 2 Tehmina Kazmi, FCPS (Pediatric Cardiology) Department of Pediatric Cardiology, University of Child Health Sciences, The Children’s Hospital, Lahore, Pakistan; 3 Munawar Ghous, (Masters Biostatistics) Department of Biostatistics and research, University of Child Health Sciences, The Children’s Hospital, Lahore, Pakistan; 4 Masood Sadiq, FRCP, FRCPCH Department of Pediatric Cardiology, University of Child Health Sciences, The Children’s Hospital, Lahore, Pakistan

**Keywords:** Cyanotic Congenital Heart Disease, Hemostatic Challenges, Thrombocytopenia, Coagulation Abnormalities

## Abstract

**Objective:**

The study aimed to assess the hemostatic complications in children with uncorrected CCHD, focusing on bleeding and thrombotic manifestations, thrombocytopenia, abnormal coagulation profiles, and deficiencies in clotting factors.

**Methodology:**

This cross-sectional study was conducted from January to November 2022 at the largest tertiary care pediatric hospital in Lahore, Pakistan. Seventy-five children aged 1-14 years with diagnosed CCHD were enrolled. Detailed clinical evaluation and laboratory investigations, including Complete Blood Count, Prothrombin Time (PT), Activated Partial Thromboplastin Time (APTT), International Normalized Ratio (INR) and clotting factors’ assays were performed. Thrombophilia testing was done in patients with history of recent stroke. Data was analyzed using SPSS version 24.

**Results:**

Among the 75 patients, the majority aged between 1-5 years (n=28, 37.3%) with male to female ratio of 1.5:1. The predominant diagnosis (n= 51,68%) was Tetralogy of Fallot (TOF). Hemostatic abnormalities included thrombocytopenia (48%), prolonged PT (56%), INR (43%), and APTT (36%). Bleeding manifestations were observed in 8% of the patients, while 5.3% had a history of stroke. Raised hematocrit was found to be significantly associated with deranged APTT. Coagulation factor deficiencies including factor V, combined factor VII and X and fibrinogen, were noted in 4% of the patients.

**Conclusion:**

The study highlights significant coagulation challenges in children with uncorrected CCHD, like thrombocytopenia and coagulation factors’ deficiencies. Enhanced understanding of these hemostatic abnormalities is crucial for better patient care in resource-constrained settings.

## INTRODUCTION

Congenital heart diseases (CHD) are the most common congenital defects with an incidence of 8-10 per 1000 live births and are the significant cause of mortality and morbidity in children worldwide.^1^ The incidence in premature infants rises to 12.5 per 1000 live births.^2^ Cyanotic congenital heart disease (CCHD) account for 25% of all children with CHD. Tetralogy of Fallot (TOF) is the most common CCHD (50% of CCHD and 5% of all CHD).^3^ In Pakistan, it is estimated that more than fifty thousand children are born with CHD, and 44 in 1000 die in neonatal age anually.^4^

CCHD if left untreated can lead to significant hemostatic challenges in children, the etiology of which is muti-factorial. Middle and low-income countries face a high global burden of CHD with significant percentage of children not receiving corrective surgery at the right time due to limited skilled human resource, fragmented ICU services, delayed presentation to the hospital and lack of awareness.^5^,^6^

Long-standing hypoxemia triggers a variety of compensatory mechanisms and makes these patients invariably prone to several complications, like, erythrocytosis, hyperviscosity, thrombocytopenia, deranged platelet aggregation, clotting factors deficiency, cerebral abscess, and cerebral embolism.^4^,^5^ In addition, the accompanying reduction in plasma volume leads to significant deficiencies in platelets, fibrinogen, and other clotting factors.^7^-^9^

Understanding these hemostatic abnormalities is crucial in improving clinical outcome of such patients.^10^ There is scarcity of data from Pakistan about spectrum of late presenting children with CCHD particularly regarding the coagulation and thrombotic problems. The aim of the study was to determine the hemostatic challenges in terms of bleeding and thrombotic manifestations, in uncorrected, late presenting patients of cyanotic congenital heart diseases.

## METHODOLOGY

It was a cross-sectional study carried out from January to November, 2022, at the Department of Pediatric Cardiology & Department of Hematology, University of Child Health Sciences (UCHS), The Children’s Hospital, Lahore. Consecutive sampling was done and 75 diagnosed CCHD patients were included.

### Inclusion & Exclusion Criteria:

Children aged 1-14 years of either sex and diagnosed with CCHD by echocardiography, angiography or CT angiography were included in this study. Children with positive viral screening for Hepatitis-B, Hepatitis-C or HIV, chronic renal and/or liver disease and with known bleeding disorder or with positive family history of bleeding were excluded. Moreover, patients diagnosed as acyanotic congenital heart disease, other causes of central cyanosis with persistent pulmonary hypertension and neonates were excluded from the study.

### Ethical considerations:

The protocol of this study was approved by institutional Ethical Review Committee [IRB#2021/490/CH.UCHS; Dated: December 11, 2021]. Informed consent was obtained from parents/guardians of all enrolled children.

### Procedure

After informed consent, history and clinical assessment, Complete Blood Count (CBC), Prothrombin Time (PT), Activated Partial Prothrombin Time (APTT) and International Normalized Ratio (INR) of all patients were performed. The coagulation factors assays were done only for those patients who had abnormal primary coagulation screen (PT, INR and/or APTT). Correction of anticoagulation: blood ratio was done for all patients having hematocrit greater than 55%, by using the following formula as per lab SOPs based on Clinical & Laboratory Standard Institute (CLSI) recommendations. Residual volume of citrate in the tube = [100-haematocrit] × [sample volume]/ [595-haematocrit in %]. For thrombophilia testing, protein C, S and Antithrombin (AT) were done only for patients who presented with stroke in past six months’ period. Blood samples were collected into two evacuated tubes containing 3.2% sodium citrate for testing of PT, APTT, Fibrinogen and other factors levels and one EDTA tube for CBC, platelet count and morphology. CBC was run on Sysmex XN/1000 analyzer. Coagulation profile including PT, APTT, Fibrinogen, other coagulation factors assays and Protein C, S and AT assays were carried out on STA Compact (Diagnostica Stago).

### Statistical Analysis:

The data was analyzed using SPSS version 24. Qualitative variables like gender, disease and coagulation factor deficincy were represented as frequency and percentage. Age, Hemoglobin, PT, APTT, INR, and platelet count were presented as mean ±SD. ANOVA and an independent samples t-test were used to compare the mean values across groups. P- value ≤ 0.05 was considered significant.

## RESULTS

The patients were divided into following four groups, A-D according to the pathophysiology of the disease.

**Table:** 

CCHD with decreased pulmonary blood flow Exclusively TOF	Group A
Other CCHD with decreased pulmonary blood flow	Group B
CCHD with Increased pulmonary blood flow	Group-C
Eisenmenger	Group D

**Fig.1:**
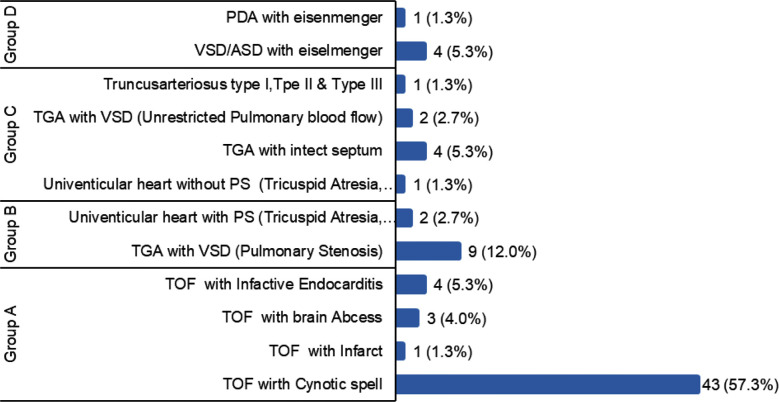
Frequency of types of cyanotic congenital heart diseases. *TOF: Tetralogy of Fallot, TGA: Transposition of the great arteries, VSD: Ventricular septal defect, PS: Pulmonary stenosis, ASD: Atrial septal defect, PDA: Patent ductus arteriosus

**Fig.2:**
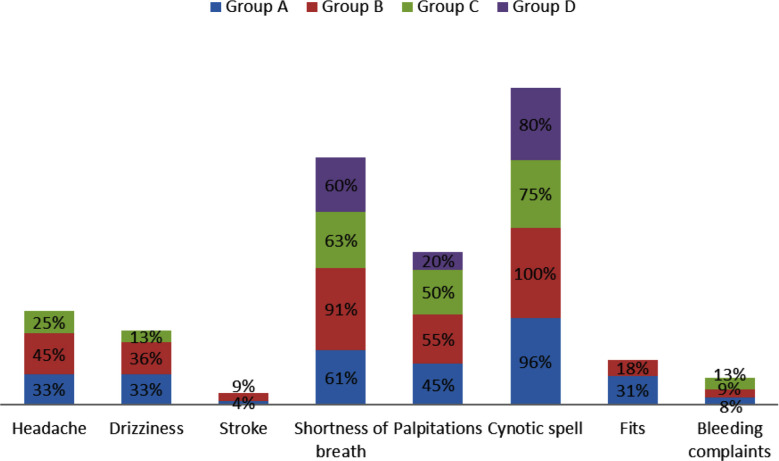
Clinical presentation of patients with cyanotic congenital heart disease.

Among the patients, the majority fell within the 1-5 years age group (37.3%), with a higher representation of males (61.3%). Tetralogy of Fallot (TOF)-Group A was the predominant diagnosis (68%). Eisenmenger syndrome was found in five patients (6.66%) (Fig.1). The most common symptom among the total number of patients, included cyanotic spells (93.3%) followed by shortness of breath. Bleeding manifestations in the form of epistaxis, gum bleed and oozing from intravenous access site were observed in 8% (6/75) patients while 5.3% have had stroke in the past six-months period in the form of hemiplegia and reversible speech deficits (Fig.2).

Hematological and Coagulation profile revealed diverse abnormalities. Polycythemia was observed in 36%, anemia in 12% and thrombocytopenia in 48% of patients. Primary coagulation screen showed prolonged PT, INR, and APTT in 56%, 43%, and 36% cases respectively. No significant difference was found in these parameters among the four groups of CCHD patients made on the basis of pathophysiology (Table-I).

**Table-I:** Hematological and Coagulation profile in patients of cyanotic congenital heart diseases.

Labs Findings		Group A	Group B	Group-C	Group D	Total
HB	Anemia	5 (10%)	3(27%)	0(0%)	1(20%)	9 (12%)
	Polycythemia	20(39%)	2(18%)	3(38%)	2(40%)	27 (36%)
	Mean±SD (g/dl)	15.32±3.47	13.25±3.21	15.25±5.07	14.70±4.74	p-value: 0.410
Platelets	Thrombocytopenia	24(47%)	3(27%)	5(63%)	4(80%)	36(48%)
	Thrombocytosis	2(4%)	0(0%)	0(0%)	0(0%)	2(3%)
	Mean±SD(x10^9^/L)	188.1±160.7	178.2±136.4	193.8±121.62	162.1±140.7	p-value: 0.419
PT	Prolonged	33(65%)	3(27%)	4(50%)	2(40%)	42(56%)
	Mean±SD (sec)	19.41±9.41	13.99±2.59	15.80±4.28	21.32±8.18	p-value: 0.625
INR	Prolonged	28(55%)	2(18%)	1(13%)	1(20%)	32(43%)
	Mean±SD	1.52±1.17	1.14±0.20	1.29±0.39	1.78±1.06	p-value: 0.581
APTT	Prolonged	18(35%)	4(36%)	3(38%)	2(4%)	27(36%)
	Mean±SD (sec)	41.35±19.54	34.28±6.63	35.70±7.14	44.80±19.72	p-value: 0.504

Among the 75 patients, 14 (18.6%) presented with complications like stroke, bleeding, brain abscess and brain infarct out of which 11 had TOF. Coagulation screen was found abnormal in patients with bleeding, brain abscess and infarct (Table-II). On the basis of HCT, patients with HCT≥50% showed significant standard deviation from their mean(p<0.05) in APTT Values (Table-III).

**Table-II:** Coagulation Abnormalities among complicated CCHD patients.

Parameters	Stroke N=4/75	Bleeding N=6/75	Brain Abscess N=3/75	Brain Infarct N=1/75
Anemia	0	1	0	0
Polycythemia	2	3	3	0
Prolonged (PT (sec))	0	4	3	1
Prolonged (INR)	0	2	1	0
Prolonged (APTT (sec))	0	4	2	1

**Table-III:** Hematocrit based group statistics of Hematological parameters in patients of CCHD diseases.

Parameters	HCT (%)		P-value
	<50	≥ 50	
	N=45	N=30	
PT (sec)	17.06±17.15	20.31±7.90	0.334
INR	1.35±1.21	1.62±0.61	0.266
APTT (sec)	35.45±14.40	46.68±19.26	<0.05^*^

Coagulation factor deficiencies were found in 3/75 patients (4%) involving four factors that is fibrinogen deficiency, Factor V deficiency and co-existent Factor X and factor VII deficiency (Table-IV). Reduced fibrinogen level of 0.71g/l was found in one patient in Group-C, with diagnosis of TGA with VSD without any bleeding phenotype. Among the deficient patient with TOF, the Factor V value was 7 IU/ml and had recurrent epistaxis and gum bleeding. Patient with low factor X value of 15 IU/ml and factor VII level of 8.1 IU/ml had complaints of spontaneous bruising. In four patients who had stroke within six-months period, Protein C, S and antithrombin (AT) levels were found within normal range.

**Table-IV:** Coagulation factors abnormalities among CCHD patients.

Factors deficiency	N=3/75 (4%)	Group A N=2/51	Group B N=0/11	Group-C N=1/8	Group D N=0/5
V deficiency	1 (1.3%)	1 (1.96%)	0	0	0
X and VII deficiency	1 (1.3%)	1 (1.96%)	0	0	0
Hypofibrinogenemia	1 (1.3%)	0	0	1 (12.5%)	0

## DISCUSSION

Our study highlights important hemostatic abnormalities in children with uncorrected cyanotic congenital heart diseases both in the form of bleeding and thrombosis. This fact reflects the intricate association between chronic hypoxemia and coagulation disorders. A prominent number of patients exhibited deranged initial coagulation screen consisting of PT, APTT, and INR, consistent with data available on coagulation abnormalities in such disorders.^9^,^11^,^12^ However, the mean values of these tests in our patients’ cohort were higher in comparison to those reported by Majiyagbe OO et al.^7^ The variance might have been reflected due to disparity in the disease severity at the time of enrollment and geographical differences. The prevalence of coagulation defects in CCHD children in our study is lower as compared to other studies from our part of Asia, which could be due to small number of their enrolled patients leading to skewed data.^13^,^14^

Thrombocytopenia (48%) was seen to be more prevalent than thrombocytosis (3%), mainly in CCHD patients with increased pulmonary blood flow. The finding is supported by the earlier studies revealing reduced bone marrow function and platelet production leading to thrombocytopenia in chronic hypoxia states and elevated pulmonary pressure.^12^,^15^,^16^ Oxygen saturation less than 85% in children with CCHD has been found to be associated with thrombocytopenia.^17^ Nevertheless, the high proportion of coagulation abnormalities along with thrombocytopenia do suggest presence of chronic disseminated intravascular coagulopathy in our study group. The finding is also highlighted in children with CHD by previous study.^7^ On the other hand, patients with Tetralogy of Fallot (TOF) predominantly showed compensatory polycythemia, pointing towards hypercoagulable state. About 50% of patients with stroke exhibited polycythemia. The findings are consistent with other studies.^12^,^18^,^19^

Although hypercoagulability risk factors were prevalent, but bleeding complications were noted in 8% of the patients. Our study corroborated the findings of the previous study showing specific clotting factor deficiencies in CCHD patients leading to bleeding tendency.^20^ We found Factor V and Factor X deficiencies in the Tetralogy of Fallot (TOF) group, correlating with clinical presentations of recurrent epistaxis and gum bleeding. Hypofibrinogenemia was found in one patient of TGA with VSD but without any bleeding complaints.

However, other patients with mildly or even moderately deranged primary coagulation screen did not show coagulation factors low enough to the level to be marked as deficient. Severe Factor V deficiency in a 12-years old CHD patient with intracranial bleeding has been described by Ozkaya H et al.^21^ The raised von Willebrand factor antigen (vWF:Ag) as a marker of endothelial dysfunction has been revealed by Ismail and co-workers.^22^ In our study, Factor X and VII deficiency in one TOF patient is indicating towards defective formation of Vitamin-K dependent clotting factors, which is likely to be associated with compromised cardiac output and chronic liver congestion as mentioned by Zabala LM et al.^17^

In the present study, CCHD patients who presented with stroke within last six-months’ time period had normal levels of Protein C, S and AT. Previous study has mentioned stroke as a common complication of CHD.^23^ The identified specific risk factors in such patients are listed as arrhythmias, cardiac failure, polycythemia, inflammation and increased platelet activation during surgery.^24^,^25^ Giglia et al have shown the reduced levels of Protein C and AT and presence of hypercoagulability linked genetic polymorphisms like factor V Leiden and prothrombin gene 20,210 in these patients.^26^

The lack of significant differences in PT, INR, APTT and platelets among the four groups suggests a shared pathophysiological mechanism driving these coagulation abnormalities, regardless of the specific type of CCHD. However, the derangement of APTT in patients was significantly associated with elevated hematocrit levels (>50%), highlighting the need for vigilant monitoring of HCT and tailored approach for these patients. Others studies have also highlighted the association of rising HCT with worsening of hematological parameters.^1^

Our study reinforces the need of comprehensive coagulation screening in CCHD patients, particularly those presenting with thrombotic or bleeding complications. The identification of specific coagulation factor deficiencies can guide targeted therapy in the form of factor replacement, which may improve clinical outcomes and reduce morbidity. Moreover, it highlights the importance of early surgical corrective procedures for these patients to prevent such complications. Since only few previous studies have focused on individual coagulation factors’ deficiencies in such patients so, this study is expected to add this aspect in the existing literature.

### Limitations

These included inability to use Thromboelastography (TEG) and Platelet function studies. Future research can focus on these diagnostic modalities to get more information about platelet functionality and coagulation cascade activation. The number of patients with different diagnosis particularly with Eisenmenger syndrome (ES) can be further enrolled and studied to highlight the role of pathophysiology of different cardiac defects on hemostatic parameters in order to develop specific monitoring strategies for such patients.

## CONCLUSION

The study underscores the significant hemostatic challenges in children with uncorrected CCHD in the form of thrombocytopenia, deranged coagulation primary screening profile and specific clotting factors deficiencies including Factor V, X, VII and fibrinogen particularly in TOF patients. The shared coagulation abnormalities across different CCHD types suggest common underlying mechanisms, while raised hematocrit can be used as a predictor for coagulopathy associated with deranged APTT. The present study can form the basis of future research studies in regard to specific coagulation factors’ deficiencies.

### Abbreviation:

**CCHD:** Cyanotic Congenital Heart Diseases

**PT:** Prothrombin Time

**APTT:** Activated partial thrombin time

**INR:** International Normalized Ratio

**TOF:** Tetralogy of Fallot

**UCHS:** University of Child Health Sciences

**AT:** Antithrombin

**HB:** Haemoglobin

**HCT:** Hematocrit

**TGA:** Transposition of the great arteries

**VSD:** Ventricular septal defect

**PS:** Pulmonary stenosis

**ASD:** Atrial septal defect

**PDA:** Patent ductus arteriosus

### Author’s Contribution:

**NS:** Conception of study, data collection and performance, manuscript writing & verification of lab investigations, review of manuscript. She is responsible and accountable for the accuracy and integrity of the work.

**TK:** Proforma design, data collection of clinical details and assessment, writing of manuscript, review of manuscript.

**MG:** Data curation and analysis, writing of manuscript, review of references.

**MS:** Study design, writing and review of final manuscript.
